# Autologous humanized mouse models to study combination and single-agent immunotherapy for colorectal cancer patient-derived xenografts

**DOI:** 10.3389/fonc.2022.994333

**Published:** 2022-09-21

**Authors:** Preeti Kanikarla Marie, Alexey V. Sorokin, Lea A. Bitner, Rebecca Aden, Michael Lam, Ganiraju Manyam, Melanie N. Woods, Amanda Anderson, Anna Capasso, Natalie Fowlkes, Michael J. Overman, David G. Menter, Scott Kopetz

**Affiliations:** ^1^ Department of Gastrointestinal Medical Oncology, The University of Texas MD Anderson Cancer Center, Houston, TX, United States; ^2^ Department of Bioinformatics & Computational Biology, The University of Texas MD Anderson Cancer Center, Houston, TX, United States; ^3^ Department of Oncology, The University of Texas Health Austin, Austin, TX, United States; ^4^ Department of Veterinary Medicine & Surgery, The University of Texas MD Anderson Cancer Center, Houston, TX, United States

**Keywords:** humanized mice, immunotherapy, colorectal cancer, pre-clinical studies, T cells

## Abstract

Designing studies of immunotherapy is limited due to a lack of pre-clinical models that reliably predict effective immunotherapy responses. To address this gap, we developed humanized mouse models of colorectal cancer (CRC) incorporating patient-derived xenografts (PDX) with human peripheral blood mononuclear cells (PBMC). Humanized mice with CRC PDXs were generated *via* engraftment of autologous (isolated from the same patients as the PDXs) or allogeneic (isolated from healthy donors) PBMCs. Human T cells were detected in mouse blood, tissues, and infiltrated the implanted PDXs. The inclusion of anti-PD-1 therapy revealed that tumor responses in autologous but not allogeneic models were more comparable to that of patients. An overall non-specific graft-vs-tumor effect occurred in allogeneic models and negatively correlated with that seen in patients. In contrast, autologous humanized mice more accurately correlated with treatment outcomes by engaging pre-existing tumor specific T-cell populations. As autologous T cells appear to be the major drivers of tumor response thus, autologous humanized mice may serve as models at predicting treatment outcomes in pre-clinical settings for therapies reliant on pre-existing tumor specific T-cell populations.

## Introduction

Cancer immunotherapy enhances the immune response in targeting cancer cells. The cytotoxic CD8^+^ T cells are among some of the predominant subsets of effectors in cancer immunotherapy that eradicate cancer cells (1). Effector cells depend on the evolution of an intra-tumoral niche (2) or tertiary lymphoid structures (3). T-cell exhaustion may also come into play that affects the tumor immune response (4). Despite the complexities of intratumoral immune responses, peripheral T cell expansion seems to help predict tumor infiltration and clinical response (5). In colorectal cancer (CRC) patients, tumors lacking activated CD8^+^ T cells predicted disease recurrence within 5 years. In contrast, a long disease-free survival was predicted for patients who had intratumoral T cells (6), thereby highlighting the importance of tumor-infiltrating immune cells in controlling the growth and recurrence of tumors. The key benefits of immunotherapy in CRC are typically limited to those with high mutational burden such as microsatellite instability-high (MSI-H) tumors (7–10). This high mutational burden is most commonly associated with an immunogenic neoantigen load that attracts tumor-infiltrating lymphocytes, consequently making them more likely to benefit from immune checkpoint inhibitors (7, 8, 11). These notions are supported by our studies that combined nivolumab and ipilimumab, which improved efficacy compared to anti-PD-1 monotherapy in MSI-H metastatic CRC, which is restricted to 5% of metatstatic CRC tumors (12). Although MSI-H early stage CRC tumor incidence is high (~15%), prognostically, these patients have a more favorable outcome compared to early stage MSS CRC resulting in a reduced percentage of MSI-H metastatic CRC (13, 14). The majority of metastatic CRC tumors are microsatellite stable (MSS) with a low neoantigen burden that is accompanied by reduced clinical benefit from immunotherapy. Pre-clinical models for evaluating MSS tumors may therefore represent an unmet need that can benefit from the development of patient centric approaches for testing broader panels of novel drugs and targeted combinations along with limiting exposure to unforeseen toxicity.

Predicting responses to combination treatments with immunotherapy requires relevant *in vivo* models that can faithfully predict tumor responses. However, pre-clinical *in vivo* models for testing the immunotherapy responses are lacking. As others and we have shown, patient-derived xenograft models (PDX) commonly use immunocompromised mice to establish a tumor growth-supportive microenvironment that more reliably reproduces clinical outcomes (15–17). These approaches depend on the immune status of the mouse strain utilized for any given study and principally interrogate the human tumor intrinsic properties of a given drug response without reflecting the human immune components. As one immunocompetent alternative, syngeneic mouse models and genetically engineered mouse models offer a robust and well-characterized system, which reflects species-specific characteristics that require careful interpretation based on macromolecular homologies and systemic immune differences. This has prompted the PDX field to develop a variety of humanized mouse models, whereby an immune deficient mouse strain is engrafted with functional human cells and tissue. These approaches reflect model specific functionality of the human immune components (18–20), but can be poorly predictive of tumor responses. Increasingly, humanized mice are used in immuno-modulation studies to interrogate interactions of the immune system with tumor cells and mechanisms of tumor escape (21–24). However, when these models utilize immune cells that do not match the tumor origin or rely on very limited HLA matching they may not manifest a clearly interpretable immune response. Mismatch of the immune system and tumor can lead to a potent allogeneic response, in which efficacy signals attributed to tumor response instead may be due to a nonspecific graft-versus-tumor response between implanted tumors and donor’s immune cells used to repopulate the immunocompromised mouse. Consequently, a genuine need exists for better models involving autologous tissues that obviate the need for HLA matching or any confounding non-personalized patient factors. To address this lack of relevant *in vivo* models this study developed a humanized mice model wherein immunodeficient mice were implanted with autologous immune cells and tumor tissue obtained from the same patient to replicate the interaction between the tumor and matched immune system *in vivo*.

## Materials and methods

### Tumor and PBMC collection

Surgically resected tumor tissue samples or biopsy samples were collected from CRC patients under a research laboratory protocol LAB10-0982 approved by The University of Texas MD Anderson Cancer Center Institutional Review Board, after patients provided written informed signed consent. In our laboratory, the samples were linked with patients but the patients remained anonymous. Confidentiality was ensured and preserved for all patient-related data. Tumor samples of metastatic CRC sites, primarily the liver, were obtained from the patients, prepared as described previously (25), and implanted subcutaneously in NSG. Tumors were harvested when they reached a volume of 1500 mm^3^. Suitable patients (MSI-H and MSS) from whom PDXs were established were identified and gave informed consent for blood draws. Blood samples were collected *via* venipuncture in tubes containing EDTA. Human PBMCs were isolated from whole blood using Lymphoprep density gradient medium (STEMCELL Technologies, # 07801) and viably frozen in fetal bovine serum with 10% dimethyl sulfoxide. Donor PBMCs were isolated from Buffy coats purchased from Gulf Coast Regional Blood Center’s research blood products.

### Animals

All mouse handling procedures were approved by the Institutional Animal Care and Use Committee. The vertebrate animals used in this study were female 4- to 6-week old NSG mice (NOD.*Cg-Prkdc^scid^Il2rg^tm1Wjl^*/SzJ, #005557) purchased from The Jackson laboratory. The mice were housed in a specific-pathogen barrier animal facility at the MD Anderson Department of Veterinary Medicine and Surgery. The veterinary care provided included feeding the animals acidified water, a Uniprim diet (Envigo, #TD.06596), and adequate crude protein, minerals, and vitamins.

Nivolumab (Opdivo, Bristol-Myers Squibb Company) was administered intraperitoneally every 5 days at 20 mg per kg body weight for 25 days. Regorafenib (#HY-10331, MedChemExpress LLC) was administered orally every day at 10 mg per kg body weight for 25 days.

### Humanized PBMC model

Human PBMCs (~4 to 5 x 10^6^) were injected into unconditioned NSG mice to generate the humanized PBMC model. Human PBMCs were suspended in sterile phosphate-buffered saline at a density of 4 × 10^6^ cells per 0.1 ml and injected intraperitoneally using 1-cc tuberculin syringes with 25-g × 5/8-inch needles (26). Similar engraftment rates were observed with intraperitoneal and intravenous PBMC injections. NSG mice received patient PBMCs that were matched with the PDXs implanted into them or donor PBMCs that were not matched. No HLA typing was done on donor blood samples. Flow cytometric analysis was performed to evaluate human cell engraftment in mice at about 2 weeks after the injections. Mice with human CD45^+^ cell percentages ≥1% were enrolled into the experimental arms. The mice were monitored daily for any health concerns after human cell engraftment. Time to GVHD symptoms and mortality were determined using time-to-event analyses. Depending on the tumor growth rates, MSI-H or MSS tumors were implanted subcutaneously into mice within the time frame of human cell engraftment as described above. Tumor fragments (~10-30 mm^3^) were cut using a scalpel, coated with 50-100 µL of Matrigel, and implanted subcutaneously in the flank regions of the mice under anesthesia. On average, three to six mice with bilaterally implanted tumors were enrolled in each experimental arm. Tumor growth was recorded every 3 to 4 days and tumor volume was calculated using the formula V = ½ (Length × Width^2^).

### Flow cytometry

Initial human cell engraftment in the study mice was done by collecting ~100 μL peripheral blood from all mice to be tested along with a blood sample from a non-engrafted mouse to be used as a negative control. Blood cells were stained with fluorochrome-conjugated antibodies. Mouse CD45-PerCP antibody was used for gating out murine leukocytes. This was followed by red blood cell lysis using BD FACS lysing solution and sample fixation for flow cytometric analysis to determine the percent engraftment in each mouse. For end point analysis of different immune cell populations, mouse blood was collected *via* cardiac puncture and processed similary. Tumors were extracted from mice, minced and enzymatically digested to generate a single cell suspension and processed for flow cytometric analysis. Single –cell suspensions from spleen, bone marrow were also prepared using standard procedures. Antibodies used for flow cytometric analysis were purchased from BD Biosciences (**Supplemental Table 1A**). All samples were run on a Gallios flow cytometer (Beckman Coulter) and data analysis was carried using FlowJo software.

### Immunohistochemistry and immunofluorescence multiplex staining

PDX tumor samples were collected and fixed in 10% neutral buffered formalin and processed routinely for histopathology. Tissues were embedded in paraffin and sectioned at 4 µm. Multiplex staining was performed using Opal 4-Color IHC Automation kit plus opal 620 (Akoya Biosciences) on a Leica Bond RXm autostainer. See antibody tables below. After staining, slides were rinsed in DI water and Vector TrueVIEW Auto fluorescence Quenching Kit was used per manufacturer’s instructions. Slides were hand-coverslipped and imaged at 20x using a Leica Versa 8 fluorescent digital scanning microscope system. Digital slides were accessed through Leica eslide manager and opened in Imagescope software. Leica digital image analysis software was utilized for quantification. A cellular immunofluorescence algorithm was tuned for each panel. Quantitative data was exported into excel spreadsheets. Antibodies used for tissue staining are included in **Supplemental Table 1B**.

### Multiplex cytokine analysis

Multiplex Cytokine analysis of mouse plasma was performed using Bio-Plex™ 200 Instrument (Bio-Rad Laboratories). Mouse blood was drawn into BD collection tubes containing citrate as an anticoagulant, and then inverted several times to mix. Blood samples were then centrifuged at 1000xg for 15 min at 4^0^C. Plasma was transferred into clean 1.5 mL tubes and centrifuged again at 10,000xg for 10 min at 4^0^C. Next, plasma samples were diluted in Bio-Plex diluent (Bio-Rad Laboratories). Human cytokines in mouse plasma samples were detected using a Bio-Plex Pro™ Human Cytokine 27-plex Assay (#M500KCAF0Y; Bio-Rad Laboratories). Plasma samples obtained from mice that did not receive any human PBMCs or PDX implantations were also analyzed to ensure that the human antibodies did not cross-react with mouse cytokines. Human plasma samples were used as positive controls. All samples were prepared and run according to the manufacturer’s recommended instructions. Analysis was carried out using Bio-Plex Manager™ Software (Bio-Rad Laboratories).

### TCR repertoire

The sequencing data for the human PBMC samples and mouse PBMC, tumor and spleen samples were analyzed using a Human reference sequence. RNA sequencing data were analyzed using a SMARTer Human TCR α/β Profiling Kit (Takara Bio). FASTQC was used to assess the quality of the data. Xenome was used to classify the sequencing data as human or mouse data. Human sequence data was only used for further analysis. MiXCR was used to align sequencing data and assemble clonotypes for the TCR repertoire. The clones corresponding to TRA and TRB chains were quantified separately. Downstream analysis of the clonotypes was performed using VDJ-tools. Analysis was done in the R computing language (version 3.6.0). Data is included in **Supplemental Table 2**

### Statistics

Analysis of data was performed using Prism software (version 8.0; GraphPad Software). Data in different groups were analyzed using an unpaired Student’s t-tests, ANOVA, or unpaired nonparametric tests (Mann-Whitney Two Sample Test) between different groups. *P* values less than 0.05 were considered significant. All tumor volume change and body weight data were summarized using means ± SD. Probability of survival was calculated using the Kaplan-Meier method.

*CRC PDX details.* Details of PDX models used in this study are included in **Supplemental Table 3**

## Results

### Characteristics of the humanized mouse model

**Figure 1:**
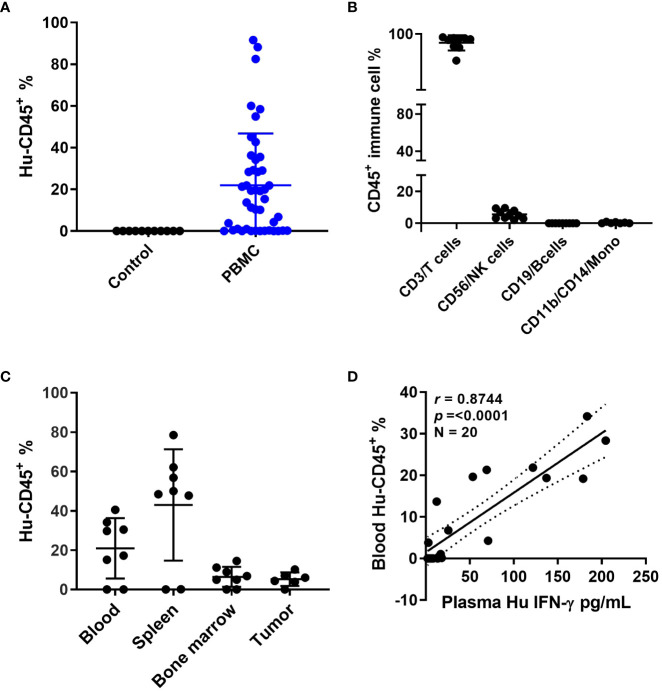
Humanized mouse development and characterization **(A)** Human CD45^+^ cell% engraftment in mice 5 weeks after injection of human PBMCs. **(B)** Different immune cell percentages in mouse blood 5 weeks after PBMC injection. **(C)** Human CD45^+^ cell percentage in different mouse tissues. **(D)** Correlation between human CD45 cell percentage in mouse blood and human interferon (IFN)-γ levels in mouse plasma.

Patient derived xenografts were generated from CRC patient tumor biopsies or surgical samples as the first required step in establishing our patient-specific humanized model. In considering another unique aspect of this particular model, we also collected blood from the same patients to match the source of immune cells required for establishing patient-specific autologous humanized mice. Human PBMCs injections were performed by multiple cell numbers and delivery routes, all of which achieved successful engraftment. The approach ultimately selected for routine use was four million PBMCs that were recovered from cryopreservation and injected by intraperitoneal delivery (data not shown). The percentage of human PBMCs as a function of human CD45^+^ (hCD45^+^) cells in mouse blood at 3-5 weeks reached ~ 50% compared to the uninjected controls (**Figure 1A** and **Supplemental Figure 1A**), these hCD45^+^ engraftment frequencies varied in a time- and donor–dependent fashion. Within the hCD45^+^ cell population, we observed that CD3^+^T cells were the predominating subpopulation in the mouse circulation along with a limited number of CD19^+^B, CD56^+^NK and CD11b^+^/CD14^+^ myeloid cells remaining after the first week of PBMC injections (**Figure 1B** and **Supplemental Figure 1B**). The highest number of human CD45^+^ immune cells were found in the mouse spleen followed by the mouse bone marrow and then the implanted PDXs (**Figure 1C**). The distribution of human immune cells in circulation over a seven-week period as defined by FACS analysis of CD45^+^ cells peaked at 5 weeks and leveled off at 7 weeks (**Supplemental Figure 1A**). The human CD3^+^T cell subpopulation represented nearly 100 percent of all cells found in the mouse circulation represented as a percentage of the total CD45^+^ population with B, NK and myeloid cells ranging from near zero to 8 percent of CD45^+^ cells (**Supplemental Figure 1B**). Human CD3^+^ immunofluorescent staining immune cells were widely dispersed throughout in the patient xenograft of the PBMC injected but not the uninjected control mice (**Supplemental Figure 1C**). We also observed human B cells, and macrophages in the implanted PDXs, although in limited numbers. To determine if these engrafted human immune cells had any anti-tumor activity in the implanted PDXs, we analyzed the tumor growth curves for PBMC-injected versus non-PBMC injected mice and observed that these cells did not impact tumor growth by themselves (**Supplemental Figure 1D**). Mouse plasma levels of human interferon gamma revealed a direct correlation with the injection of human PBMC as a function of human CD45^+^ cells present (**Figure 1D**). Other human cytokines levels in mouse plasma also substantially correlated with hCD45^+^ cell engraftment, indicating that they are active and functional within a murine xenographic hematologic environment (**Supplemental Figures 1E–H**).

### Difference in response between autologous and allogeneic models

**Figure 2:**
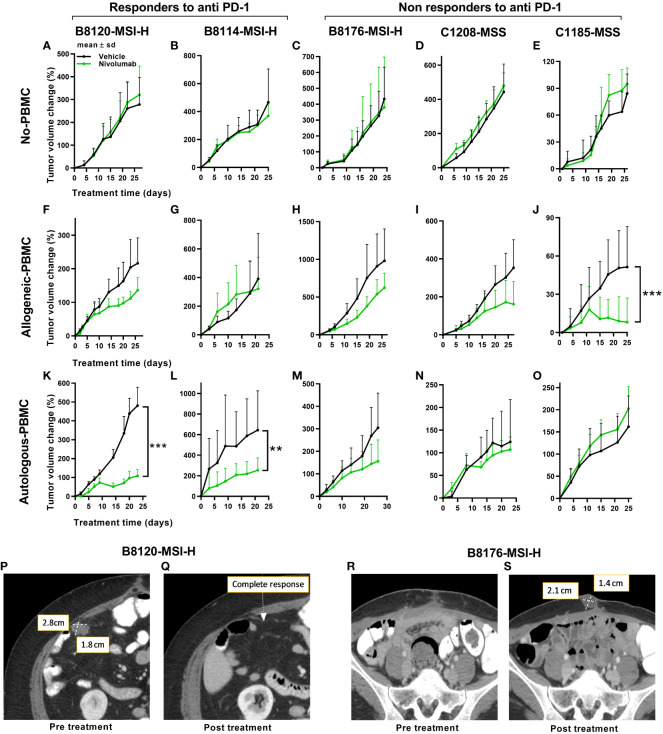
Tumor responses to anti-PD-1 therapy under non-humanized, allogeneic, and autologous conditions. Tumor volume change [ΔV_%_=100*(V_t_-V_0_)/V_0_] plots are shown here. **(A–E)** Results of anti-PD-1 therapy for five PDXs under non-humanized conditions (n=6-9 tumors/arm). **(F–J)** Results of anti-PD-1 therapy for five PDXs under allogeneic conditions (n=6-9 tumors/arm). **(K–O)** Results of anti-PD-1 therapy for five PDXs under autologous condition (n=6-9 tumors/arm). The green lines and dots represent treatment with nivolumab at 20mpk Q5D. **(P, Q)** Scans of the patient corresponding to the B8120 model before **(P)** and after **(Q)** immune checkpoint therapy. **(R, S)** Scans of the patient corresponding to the B8176 model before **(P)** and after **(Q)** immune checkpoint therapy. *P* values ≤ 0.001 were represented as ***. and P values ≤ 0.01 were represented as **.

To address the possibility that differences in immune response exist between autologous and allogeneic sample pairings we generated two different series of genetic stability models. To that end, we compared the tumor volume changes under allogeneic (un matched donor immune cells) and autologous (matched patient immune cells) conditions in five PDX models with anti-PD-1 therapy. Three were MSI-H and two were MSS models that were done in parallel using patient PBMCs or donor PBMCs. To better understand any potential therapeutic connections of our mouse models to the immunotherapy responses of our patients, PDXs were subcategorized further into anti-PD-1 responders or non-responders (**Figure 2**). Following this stratification, two of the MSI-H tumors segregated into the responding group (B8120 & B8114) as might be expected, whereas one of the MSI-H (B8176) segregated with the non-responding MSS tumors (C1208 & C1185). When all five sub-stratified PDXs remained void of any human PBMCs, no significant difference was observed in growth rate or volume between anti-PD-1 treated and untreated tumors (**Figures 2A–E**). This finding also ruled out the impact of any human immune infiltrates that may be present within the passaged PDXs. **Figures 2F–J** show the five PDXs under allogeneic conditions subjected to anti-PD-1 therapy. **Figures 2K–O** show the five PDXs under autologous conditions also subjected to anti-PD-1 therapy. Any effects on tumor growth from allogeneic PBMCs was far less predictable and was less profound in anti-PD-1 treated mice. Unexpectedly, however, MSI-H-PD-1 responsive tumors (**Figures 2F, G**) were less responsive to anti-PD-1 in allogeneic conditions. Furthermore, in some cases the responses seen in allogeneic models (**Figure 2J**) contradict with that seen in a patient. These results are consistent with the hypothesis that allogeneic sources produce nonspecific immune responses.

In contrast, when patient PDXs were matched with autologous PBMCs followed by anti-PD-1 therapy, the MSI-H tumors B8120 and B8114 showed the greatest overall response consistent with the corresponding patients response (**Figures 2K, L**). Similarly, the response to anti-PD-1 therapy in autologous models generated from non-responding patients was more consistent with the response seen in patients (**Figures 2M–O**). The anti-PD-1 treated PDX taken from a non-responding MSI-H patient, B8176, corresponded more accurately to what occurred in the CRC patient donor who progressed on anti-PD-1 therapy. Also and more consistent with therapeutic response to anti-PD-1 therapy commonly seen in MSS patient donors who progress on anti-PD-1 therapy, the autologous condition showed no response when similar treatments were performed in our humanized mouse models (**Figures 2N, O**). Our results suggest that allogeneic conditions do not represent the outcome of treatment as effectively as autologous models. Body weight changes were minimal in all mice, including non-humanized (**Supplemental Figure 2A**), allogeneic (**Supplemental Figure 2B**), and autologous condition (**Supplemental Figure 2C**) suggesting that this approach was well tolerated. Human immune cell engraftment percentages were also high based on hCD45^+^% presence in the peripheral blood before and after treatment are shown in **Supplemental Figures 2D, E** for allogeneic and autologous models respectively. Tumor immune infiltrates after anti-PD-1 therapy are shown in **Supplemental Figures 2F, G** for allogeneic and autologous models respectively.

### Proof-of-concept study using humanized mouse models of CRC

In further support of autologous PBMC and PDX humanized mice being more representative of patient responses, anti-PD-1 treatment of two MSI-H tumors in mice more accurately reflected the before and after patient response by MRI in MSI-H responders (**Figures 2K–L**) compared to MSI-H non-responders (**Figures 2M–O**). Our results show a responding MSI-H PDX model B8120 (**Figure 2K**) and a non-responding MSI-H PDX model B8176 (**Figure 2M**) to anti-PD-1 therapy in an autologous setting. Radiographic scans of the patient corresponding to the B8120 model before (**Figure 2P**) and after (**Figure 2Q**) immune checkpoint therapy showing complete response, along with the scans of the patient corresponding to the B8176 model before (**Figure 2R**) and after (**Figure 2S**) immune checkpoint therapy showing a progressive disease are included here to highlight the results from our autologous mouse models and how they compare to that of the patients.

### T-cell receptor repertoire in the humanized mouse model of CRC

**Figure 3:**
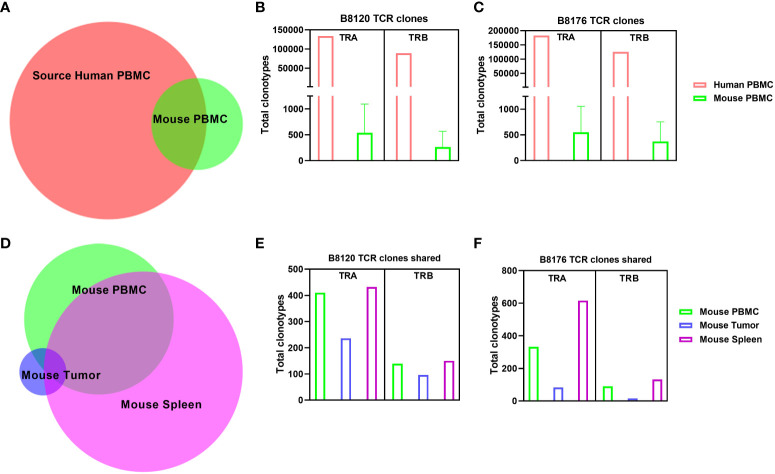
Comparison of human TCR sequences in human and humanized mouse samples **(A)** The overlap of clonotypes between human PBMCs and mouse PBMCs. **(B, C)** The number of TRA and TRB clones in human and mouse blood samples in the models B8120 **(B)** and B8176 **(C)**. **(D)** The extent of overlap of clones in different mouse tissues. **(E, F)** Bar charts of the B8120 **(E)** and B8176 **(F)** clonotypes that are common in human PBMCs injected into mice and mouse tissues after injection.

To examine how the T-cell receptor (TCR) repertoire is altered when human T cells are injected into a murine xenographic system under autologous conditions, we performed total cross-species TCR α/β profiling to compare the clonotypes of human PBMCs in mouse tissues before injection and when dissociated from mouse tissues for two separate human tumor xenografts (B8120 and B8176) after injection. **Figure 3A** shows the overlap of clonotypes observed between human and mouse PBMCs. The number of TCR α chain (TRA) and TCR β chain (TRB) clones in human and mouse blood samples for models B8120 and B8176 revealed similar distributions (**Figures 3B, C**). When comparing clonotypes between mouse blood, tumor, and spleen samples only a small clonal population occurred between all three mouse tissues (**Figure 3D**). Further post-injection analysis revealed similar fluctuations between B8120 and B8176 with the highest preponderance of engraftment occurring in the spleen for both TRA and TRB clonotypes (**Figures 3E, F**). We observed that although the overall TCR diversity of the implanted PBMCs and persisting PBMCs in mouse samples decreased, homogeneity (overlap) of the TCR repertoire between human PBMCs and mouse tissue samples was maintained. The rearrangement patterns for the TCR chains within the TRA clones are represented in circos plots in **Supplemental Figures 3A–D** and those within the TRB clones are represented in circos plots in **Supplemental Figures 3E–H** for model B8120. Similarly, circos plots of these rearrangement patterns in model B8176 are shown in **Supplemental Figures 3I–L** (TRA) and 3M-3P (TRB). We observed that clones that were infrequent or undetermined in human PBMC samples appeared to be outgrown in mouse tissue samples. Decreased TCR diversity in mouse samples along (shown by fewer circumferential designations) with low overlap with human PBMC clonotypes in these samples suggested that a fraction of the T cells present at low frequencies in human blood expanded in mouse tissues. This might be the case in the complementarity-determining region TRBV5-1(orange spoke)/TRBJ1-2 (dark blue spoke) clonotype for example that is present in in all B8120 and B8176 samples. In contrast, complementarity-determining regions TRAV30/TRAJ53 were prominently present in mouse PBMC, mouse spleen and B8176 PDX tumors that were generated from MSI-H/anti-PD-1 unresponsive donor samples. The disparity of complementarity-determining regions was greater in B8120 generated from MSI-H/anti-PD-1 responsive donor samples, with no distinct complementarity-determining regions clearly observed potentially reflecting a higher neoantigen load.

### Combination treatment strategies for MSS CRC PDXs

**Figure 4:**
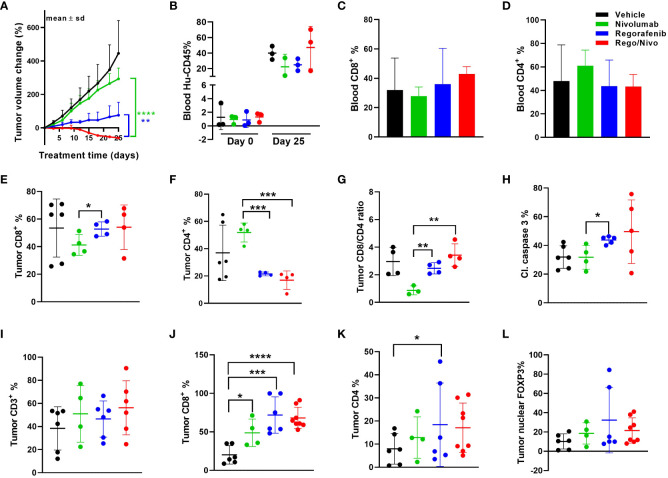
Results of anti-PD-1 and regorafenib combination therapy for MSS CRC model **(A)** Tumor volume change [ΔV_%_=100*(V_t_-V_0_)/V_0_] plots for the C1221 model engrafted with autologous PBMCs and given treatment with nivolumab and regorafenib (n=4-8 tumors/arm). **(B)** Human hCD45^+^ cell percentages in mouse blood before and after the experiment. **(C, D)** Percentages of CD8^+^ **(C)** and CD4^+^ **(D)** T cells (percent of CD45^+^ cells) in mouse blood at the experimental end point. **(E, F)** Tumor CD8^+^ **(E)** and CD4^+^ **(F)** T-cell percentages determined using flow cytometry (percent of CD45^+^ cells). **(G)** Tumor CD8^+^:CD4^+^ T-cell ratios. **(H)** Cleaved caspase 3^+^ cell percentages in tumors assessed using immunohistochemistry. **(I)** Results of Immunohistochemical analysis of CD3^+^ T cells in tumors. **(J**, **K)** CD8^+^ **(J)** and CD4^+^ **(K)** T-cell percentages in tumors. **(L)** Nuclear FOXP3^+^ cell percentages in tumors assessed using immunofluorescent staining. The green lines and dots represent nivolumab-based treatment (every 5 days, 20 mpk), the blue lines and dots represent regorafenib-based treatment (everyday, 10 mpk), and the red lines and dots represent combination treatment. All *P* values ≤ 0.05 were represented as *, *P* values ≤ 0.01 were represented as **, *P* values ≤ 0.001 were represented as ***, and *P* values ≤ 0.0001 were represented as ****.

Because we saw synonymous responses to anti PD-1 therapy in CRC patients and their PDXs in humanized mice in the autologous setting, we next explored the viability and activity of combination treatment strategies in MSS CRC PDX models. The combination of regorafenib and nivolumab has demonstrated clinical activity, albeit modest, in MSS CRC patients (REGONIVO trial) (27, 28), whereas treatment with either agent alone has not resulted in tumor regression. Similar activity reported in abstract form has been seen with other combinations of VEGFR tyrosine kinase inhibitors and PD-1/PDL1 inhibition (29, 30). Pre-treatment and post-treatment biopsies for patients in the REGONIVO study demonstrated modulation of the percentage of T-cell infiltrates. To determine whether our models can recapitulate these clinical findings, we tested regorafenib in combination with nivolumab in the MSS CRC PDX model C1221 under autologous conditions and observed that the combination was more efficacious in reducing tumor volume than was either agent alone (**Figure 4A**). The human immune cell percentages in mouse blood before and after treatment are shown in **Figure 4B**. The percentages of CD8^+^ and CD4^+^ T cells in the blood of mice did not appear to vary with different treatments (**Figures 4C, D**). Assessment of tumor CD8^+^ and CD4^+^ T-cell percentages using flow cytometry revealed a trend toward a higher CD8^+^ T-cell percentage (**Figure 4E**), a significantly lower CD4^+^ T-cell percentages (*P*=0.0004, **Figure 4F**) and a higher CD8^+^:CD4^+^ T-cell ratio (*P* = 0.004, **Figure 4G**) in the combination arm than in the nivolumab-alone arm. CD8^+^ and CD4^+^ T-cell numbers per gram of tumor weight are shown in **Supplemental Figures 4A, B**. We observed increased total cleaved caspase 3 positivity in the regorafenib-alone (significantly, *p*=0.017) and combination (*P*=0.174, **Figure 4H**) groups than in the nivolumab-alone group. We also analyzed tumor CD3^+^, CD4^+^, and CD8^+^ T-cell and FOXP3^+^ cell percentages *via* immunofluorescence staining (**Figures 4I–L**). Furthermore, our analysis shows how the total cell percentage was distributed within the intratumoral (**Supplemental Figures 4C–F**) stromal (**Supplemental Figures 4I–L**) and tumor compartments. We observed a slight down modulation in the distribution of CD4^+^ T-cells (**Supplemental Figure 4K**), and nuclear FOXP3^+^ cells (**Supplemental Figure 4L**) within the stromal compartment of the tumors in the combination treatment arm. We also saw a reduction in the distribution of the number of CD4^+^ T-cells with combined treatment with regorafenib and nivolumab in this model, which was consistent with the findings in reported studies (27).

We further explored other immune cell markers with multiplexing immunofluorescence to identify other immune cell infiltrates in the PDXs. Notably; we identified CD20+ B cells and some myeloid cells in the mouse tumors even though the percentages of these cells in the blood of the mice were at most minimal. In the case of B cells, we observed a trend toward a higher percentage (**Supplemental Figure 4M**) and a significantly lower percentage of macrophages (*P*=0.013, **Supplemental Figure 4N**) in the stromal compartment in the combination group than in the nivolumab-alone group. **Supplemental Figure 4O** shows that combining anti-PD-1 therapy with a reduced regorafenib dose (50% lower than the clinically tested dose) resulted in a markedly lower tumor volume than did single-agent treatments in the MSS CRC PDX model C1211.

### Graft-versus-host disease onset and window of treatment in PBMC humanized mice

**Figure 5:**
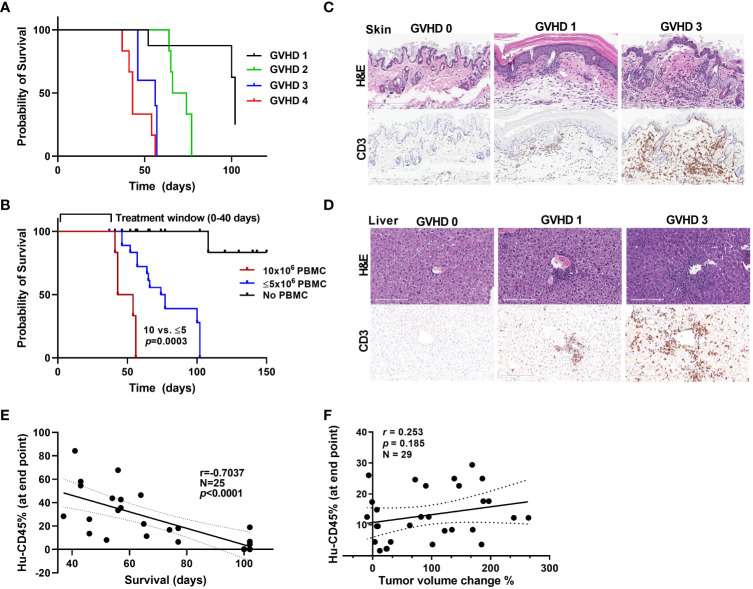
GVHD onset and the window of treatment **(A)** Survival curves for mice based on their GVHD scores. **(B)** Survival curves for mice that received different numbers of PBMCs. **(C, D)** Stains showing the level of infiltration of human CD3^+^ cells into mouse skin **(C)** and liver **(D)** samples at different stages of GVHD. **(E)** The correlation between the hCD45 cell percentage in mouse blood at 5 weeks after injection of PBMCs and survival time in mice. **(F)** The correlation between the hCD45 cell percentage in mouse blood at the experimental end point (at 5 weeks after injection of PBMCs) and percent tumor volume change in anti-PD-1 antibody-treated mice.

Though this humanized PBMC mouse model provides co-clinical experimental advantages when compared with humanized CD34^+^ model, with quick engraftment times and requiring few milliliters of patient blood, it does present with the limitation of graft-versus-host disease (GVHD) onset as a function of enrichment in engrafted human T cells. As in other transplant models, onset of GVHD in the model we used in the present study has been attributed to the expansion of human T cells that are reactive to murine tissues and actively target mouse cells (31–33). We used a scoring system to assess the extent of GVHD in our model. The scoring criteria for GVHD that we used were based on weight loss, hunched posture, poor fur texture, diminished skin integrity, and diarrhea based on previous studies (34). **Figure 5A** shows survival curves for mice based on their GVHD scores. Acceptable body weight loss and other symptoms suggested that the window of treatment could be within 45-50 days after PBMC injection, with mice with higher GVHD scores having higher mortality rates. We observed considerable differences in the survival curves for mice that received 1-5 million PBMCs and those that received 10 million PBMCs (**Figure 5B**), suggesting that injecting low cell numbers can delay the impact of GVHD in these mice. GVHD progression in mice skin and liver with human CD3+ cell infiltration are shown in **Figures 5C**, **D**. We also observed that as the percent human cell engraftment in these mice increased their survival times decreased (**Figure 5E**), possibly due to a predominance of T cells in these mice leading to the onset of GVHD and manifestations of cytokine storm (35). Because GVHD can have a significant impact on mouse health, we sought to determine if GVHD by itself causes changes in tumor volume in these mice. **Figure 5F** shows that the human CD45^+^ cell percentages in mouse blood were not correlated with their tumor volume change percentages. This suggests that onset of GVHD alone does not cause a reduction in tumor volumes in anti-PD-1 antibody treated mice, attributing any changes seen in tumor volume to the anti-tumor activity of T cells.

## Discussion

The use of humanized mouse models for immuno-oncology research is emerging as a way to help interrogate and manipulate human immune function in a complex immune compromised organism. This area of research has benefitted from the development of a variety of immunocompromised mouse strains genetically engineered to support specific immune function or immune cell type behavior (36, 37). Studies reported in the literature have used mouse models generated from human CD34^+^ cells and PBMC sources to evaluate tumor-immune responses with different immunotherapeutic agents (38–41). Humanized mouse models on a BALB/c-Rag2^null^Il2rγ^null^SIRPα^NOD^ 9B (BRSG) background demonstrated human immunity and PD-1-expressing T cells, thereby, providing the basis for pre-clinical immunotherapy studies (23). NSG-beta2m^(-/-)^ that lack *Prkdc* gene, the X-linked *Il2rg* gene and the *B2m* gene have also been used to support PBMC growth to test Bintrafusp alfa (M7824) a bifunctional fusion protein composed of the extracellular domain of the TGF-betaRII to function (42). We have tested multiple immunocompromised strains in support of our patient PBMC growth to settle upon the use of unconditioned NSG (NOD.*Cg-Prkdc^scid^Il2rg^tm1Wjl^*/SzJ) mice as balanced against closely monitored GVHD (34), which provided a very workable window of treatment of 45-50 days. The advantage of using PBMCs to generate a humanized mouse model over other sources is that this model enables rapid engraftment and enriches for human CD3^+^ T-cell engraftment, which is ideal for T cell-mediated tumor regression studies (43, 44). However, most of the currently available humanized mouse models of cancer have some degree of allogeneic response due to partial or complete mismatch of the implanted human tumors and injected immune cells. This led to our development of an autologous humanized mouse model of CRC using patient PBMCs to address this gap.

In our humanized PBMC mouse model, engrafted T-cells were functional, with the ability to produce cytokines. In addition, the T-cell clonotypes originally seen in human PBMC sources were retained in this mouse model, and our TCR profiling showed that some T-cell clones are shared by mouse blood and PDXs whereas others are expanded only in PDXs, suggesting that these clonotypes could be specific against tumors. Clonotype-driven responses are critical to understanding the underlying biology of immune responses (45). This seems to be particularly true of cancer immunobiology whereby TCR profiling can help determine the differences between immune tissues, circulating or tumor infiltrating lymphocytes and responses to checkpoint inhibitor responses. In one study TCR β chain complementarity-determining regions in non–small cell lung cancer (NSCLC) treated with checkpoint inhibitors can help determine the degree of overlap of the TCR repertoire between tumor-infiltrating lymphocytes and circulating PD-1+CD8+T cells to determine the shared TCR clones. The resulting TIR index correlated with response and survival outcomes of anti–PD-(L) 1 treatment (46). Other complementarity-determining regions TRBV2/TRBJ1-2 and TRBV2/TRBJ1-1 have been reported before as potential prognostic markers in the case of papillary thyroid cancers (47). These studies focused on TCR-B signatures, which may be more reflective of the infiltrating T-cell clonotypes, much like our results showing complementarity-determining region TRBV5-1/TRBJ1-2 clonotype that is present in all B8120 and B8176 samples. Therefore, this T cell immune recognition could be a potential mechanism by which tumor growth reduction occurs in such autologous models. Moreover, the diversity of TCRs decreased in the mouse samples with expansion of clones that were present at lower frequencies in the human PBMC samples. These results are similar to reductions in TCR diversity 14 days after transplantation in comparison to TCR diversity at the initial infusion of allogeneic PBMCs that was dependent on the HLA status of the mouse background strain, either an NSG or an NSG-HLA-A2/HHD (48). Allogeneic PBMCs in these studies were paired with luciferase-expressing THP-1 cells to evaluate graft-versus-leukemia soluble tumor effects and revealed a greater anti-tumor effect on the NSG-HLA-A2/HHD vs the NSG mouse strain (48). In our case the unconditioned NSG mouse background did not seem to influence our autologous pairings, which maintained the PD-1 response profile exhibited by the patient tumors.

Most current approaches to humanization of mice and models used for immuno-oncology are limited by access to patients and matching immune cells. As a result, the models rely on very limited human leukocyte antigen matching for CD34^+^ cell implantation at considerable expense and with limited reproducibility. Moreover, the major drawback of using some of these models may arise from the allogeneic responses resulting from the unmatched human immune cells and human tumors, which are by their very nature more difficult to predict than autologous responses. When we performed a head-to-head comparison between matched patient PDX and autologous PBMCs versus unmatched allogeneic tissues in humanized mice the allogeneic responses were less predictable. When we sub stratified our findings based on donor responses to checkpoint inhibitors, autologous models with anti-PD-1 therapy more closely reflected the patient responses. These comparable responses in our autologous PDXs and their corresponding patient tumors suggests concordance and reliability of the model in predicting cancer responses to immunotherapy. We have previously observed concordance between responses in pre-clinical PDX and clinical trial observations in both immune and non-immune approaches (23, 25). In the present study, we observed that this concordance was lacking in our allogeneic models. Our proof-of-concept results regarding anti-PD-1 therapy in the MSI-H and MSS PDXs demonstrate that tumor responses to this therapy differ in the allogeneic and autologous settings. The responses seen in most PDX models under allogeneic conditions do not reflect those seen in the corresponding patients. Using PBMCs isolated from CRC patients, we showed in the present study that our autologous models could capture responses based on the patients’ immune potential, T-cell recognition status and clinical responses that can potentially serve as a personalized co-clinical approach or tumor subclass analytical tool.

As the majority of CRCs are MSS and fail to show any clinical benefit with immunotherapy, they are ideal for generation of models for use in pre-clinical trials of new agents administered alone and in combinations. Modulation of immuno-suppressive cells is being explored to overcome the limited efficacy of immunotherapy for MSS CRC. Regorafenib is a multi-kinase inhibitor that targets various receptor tyrosine kinases, and research has shown that it can reduce the number of immunosuppressive regulatory T cells and tumor-associated macrophages (27, 28). This led us to explore combination anti-PD-1 therapy with regorafenib in our humanized models with MSS CRC PDXs. Similar to early results reported with this combination in patients and similar results recently presented for other VEGFR tyrosine kinase inhibitors and anti-PD-1 combinations, we observed considerably greater tumor volume reduction with the combination of regorafenib and nivolumab than with either agents alone in our models C1221 and C1211 (29, 30, 49). In addition, flow cytometry and immunofluorescent analysis of tumor samples demonstrated a significant reduction in the percentage of CD4^+^ T cells along with Arginase-1^+^ cells/macrophages in the tumors in the combination treatment arm than in the nivolumab-alone arm. This is consistent with paired biopsy findings from the clinical trial, further highlighting the applicability and benefits of such autologous humanized mouse models in understanding how immune cells are modulated by a given therapy. Future studies focusing on combining VEGF and anti-PD-1 combinations using these mouse models can provide insights on tumor immune microenvironment changes.

One of the limitations of the present study is the use of patient PBMCs to populate the mouse immune system, where most of the cells are already differentiated and mature. Others have noted that while differences may be observed in some myeloid and B-cell lineages using NSG-beta2m^(-/-)^ mice at the time of injection, appropriate freeze/thawing of adoptively transferred cells prior to injection does not appear to change survival or phenotypes of T-cells post engraftment (42). Similarly, our method using unconditioned NSG to support lymphoprep prepared PBMCs from whole blood and cryopreserved in contrast to using patient-isolated CD34^+^ cells, predominantly enriches in T cells and is less supportive of other immune cell types in the circulation. However, our results showed that other immune cell types could also engraft and infiltrate tumors. Furthermore, we observed that these infiltrated immune cell types and their numbers can be modulated with anti-PD-1 combination therapy. The infiltration of myeloid cells and their modulation in these humanized mouse tumors with therapy is a novel observation. Mouse models generated using CD34^+^ cells have proven to be beneficial in studies requiring T-cell priming and in vaccine studies, and they have the advantage of including most if not all immune cell lineages (39, 50). However, autologous modeling requires isolation of CD34^+^ cells from cancer patients through invasive procedures such as bone marrow biopsies and leukapheresis, which are not routinely feasible. Given the complexity of the procedures and safety concerns that arise in isolating hematopoietic stem cells from cancer patients, we believe that the present humanized PBMC model is the most practical option for studies of immuno-oncology. The ability to generate humanized mice with minimal peripheral blood volumes also makes it feasible. The use of newly available mouse strains with expression of human cytokines or those lacking mouse major histocompatibility proteins could potentially improve support of engrafted immune cells but our therapeutic window was adequate when balanced against autologous PDX growth. However, we are aware of the bias of the results generated from established PDXs over those models that were not established.

Another limitation of the PBMC-humanized mouse model is the development of GVHD within 5-8 weeks (51). The onset of GVHD has been attributed to expansion of the population of human CD8^+^ T cells that actively target mouse cells *via* recognition of the major histocompatibility complex I proteins on mouse cells (31–33). Our results from using different PDX models suggest that we can delay the onset of GVHD by reducing the number of PBMCs injected to generate this model. We found that immune cells from different donors have varying engraftment rates even when the same numbers of cells were injected, with some donor cells engrafting at a higher rate than others and exacerbating GVHD. To determine if a large increase in the number of human immune cells in mouse blood can cause tumor volume regression, skewing our observed results, we looked at the correlation between the hCD45^+^ cell percentage in mouse blood at the experimental end points and the changes in the tumor volumes of mice given anti-PD-1 therapy. We did not observe an obvious correlation between them, suggesting that high numbers of human cells did not cause a reduction in tumor volume. This observation that T cells are not active toward the tumor with anti-PD-1 treatment despite heavy T-cell infiltration (~60%) suggests that the T-cell responses toward the tumor are very specific.

Overall, these autologous humanized mouse models provide the opportunity to perform immediate short-term studies that can reduce the time of pre-clinical efforts with more reliable patient specific tumor responses that have the potential to better inform therapeutic options when compared with allogeneic models. These results are essential to developing and building up-on these models for future drug combination and efficacy studies. Furthermore, these PDXs were generated from late-stage metastatic CRC patients who have already undergone first- and second-line therapy. The efficacy readouts for novel therapies will indeed reflect the potential of these therapies in CRC patients whose disease does not respond to standard-of-care treatment. These models also provide considerable diversity as they originate from tumors with different mutational statuses that we expect to see reflected in a diverse patient population.

## Data availability statement

The original contributions presented in the study are included in the article/**Supplementary Material**. Further inquiries can be directed to the corresponding author.

## Ethics statement

The studies involving human participants were reviewed and approved by The University of Texas MD Anderson Cancer Center Institutional Review Board (under a research laboratory protocol LAB10-0982 for obtaining tumor and blood samples from patients). Patients provided consent for tumor and blood specimens to be used for research purposes along with the generation of PDXs. Written informed consent was also obtained from the individual(s) for the publication of any potentially identifiable images or data included in this article. The animal study was reviewed and approved by The University of Texas MD Anderson Institutional Animal Care and Use Committee (protocol #1077-RN02). All in vivo experiments utilizing PDXs were performed in accordance with institutional policies.

## Author contributions

PK: Conceptualization, validation, visualization, methodology, data recording, sample collection and processing, formal analysis, writing original draft and editing. AS: Conceptualization, validation, visualization, methodology, formal analysis, writing original draft and editing. LB: Investigation, data recording, animal care and maintenance, sample collection and processing. RA: Investigation, animal care and maintenance, sample collection and processing. ML: Conceptualization, writing original draft and editing. GM: Software, formal analysis, and methodology. MW: animal care and maintenance, and sample collection. AA: Immunostaining and data analysis. AC: Conceptualization, writing and editing. NF: Immunostaining data, software, data analysis, methodology, writing and editing. DM: Project administration, supervision, visualization, funding acquisition, writing and editing. MO: Conceptualization, resources, and supervision. SK: Conceptualization, resources, formal analysis, supervision, funding acquisition, investigation, visualization, methodology, writing original draft, project administration, and editing. All authors contributed to the article and approved the submitted version.

## Funding

The Cancer Prevention Research Training Program, Janice Davis Gordon Memorial Postdoctoral Fellowship in Colorectal Cancer Prevention at MD Anderson, supports PM. SK and DM are supported by the GI-SPORE-5P50CA221707, 5U54CA224065, MD Anderson’s Institutional Research Grant, MD Anderson’s Colorectal Cancer Moon Shots ProgramTM and The E.L. and Thelma Gaylord Foundation. AC is supported by CPRIT Scholar in Cancer Research grant RR160093 and DoD Career Development Award W81XWH-20-1-0366. This study used the Flow cytometry and Cellular Imaging Core Facility, Advanced Technology Genomics Core, and Biospecimen Extraction Facility core, which are supported in part by the National Institutes of Health through MD Anderson’s Cancer Support Grant CA016672.

## Acknowledgments

The authors would also like to acknowledge MD Anderson Cancer Center’s Research Medical Library for editing of this manuscript. Sample processing for IHC and IF multiplex immune staining was performed in MD Anderson’s DVMS veterinary pathology lab.

## Conflict of interest

SK reports stock or ownership interests in Navire and Lutris; consulting or advisory roles to EMD Serono, Merck, Holy Stone Healthcare, Novartis, Lilly, Boehringer Ingelheim, AstraZeneca/MedImmune, Bayer Health, Pierre Fabre, Redx Pharma, Ispen, Daiichi Sankyo, Natera, HalioDx, Lutris, Jacobio, Pfizer, Repare Therapeutics, Inivata, Jazz Pharmaceuticals, Roche, Navire, and Amgen; and research funding (to the institution) from Roche, EMD Serono, MedImmune, Novartis, Amgen, Lilly, and Daiichi Sankyo; and research funding (to self) from Merck, Navire, Holy Stone Healthcare, Boehringer Ingelheim, AstraZeneca/MedImmune, Bayer Health, Pierre Fabre, Redx Pharma, Ispen, Natera, HalioDx, Lutris, Jacobio, Pfizer, Repare Therapeutics, Inivata, and Jazz Pharmaceuticals. MJO: Consulting or Advisory Role: 3D Medicines, Bristol Myers Squibb, Roche/Genentech, Gritstone Oncology, MedImmune, Merck, Novartis, Promega, Spectrum Pharmaceuticals, Array BioPharma, Janssen, PfizerResearch Funding: Bristol Myers Squibb, Merck, Roche, MedImmune. Consulting: Merck, Astra-Zeneca, Takeda, Pfizer, Array, Gritstone, and Promega.

The remaining authors declare that the research was conducted in the absence of any commercial or financial relationships that could be construed as a potential conflict of interest.

## Publisher’s note

All claims expressed in this article are solely those of the authors and do not necessarily represent those of their affiliated organizations, or those of the publisher, the editors and the reviewers. Any product that may be evaluated in this article, or claim that may be made by its manufacturer, is not guaranteed or endorsed by the publisher.
