# Supplementary material for: Autologous humanized mouse models to study combination and single-agent immunotherapy for colorectal cancer patient-derived xenografts
Source: Front Oncol. 2022 Sep 21;12:994333. doi: 10.3389/fonc.2022.994333 (PMC9532947; doi:10.3389/fonc.2022.994333)
Supplement: Supplementary file 1 [file DataSheet_1.pdf]

Supplemental Data

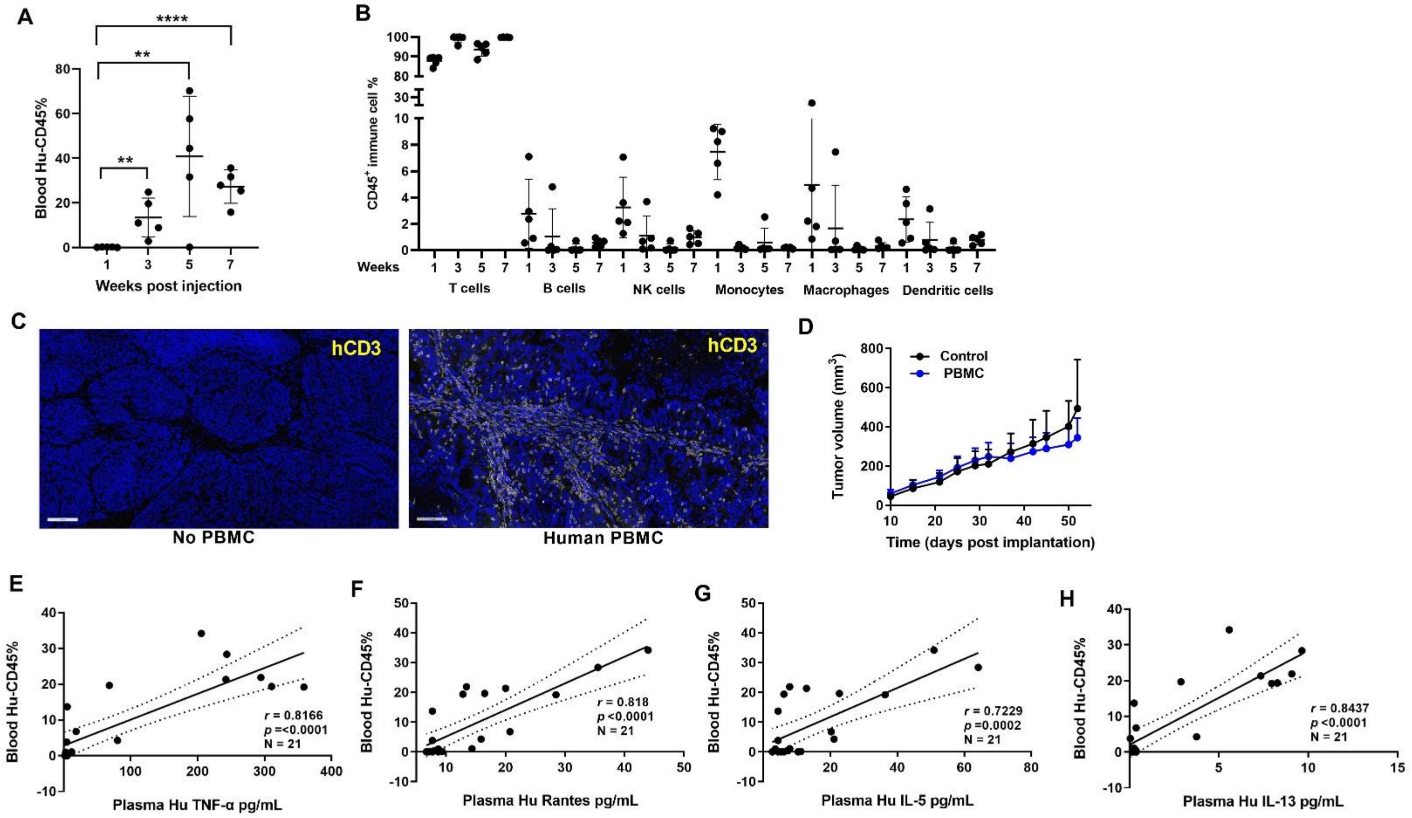

Supplemental Figure 1. **Humanized mouse characteristics**

(**A**) Changes in hCD45$^+$ immune cells over time (week 1 through week 7 post PBMC injection) in mouse blood. (**B**) Changes in sub populations of hCD45$^+$ immune cell subtypes over time in mouse blood. (**C**) hCD3$^+$ staining in a nonhumanized mouse PDX (left) and a humanized mouse PDX (right). (**D**) PDX growth curves for mice with and without injection of human PBMCs. (**E-H**) Correlation of the hCD45$^+$ cell percentage in mouse blood with human tumor necrosis factor (TNF)-α (**E**), Rantes (**F**), interleukin (IL)-5 (**G**), and IL-13 (**H**) level in mouse plasma. *P* values ≤ 0.01 were represented as ** and *P* values ≤ 0.0001 were represented as ****.

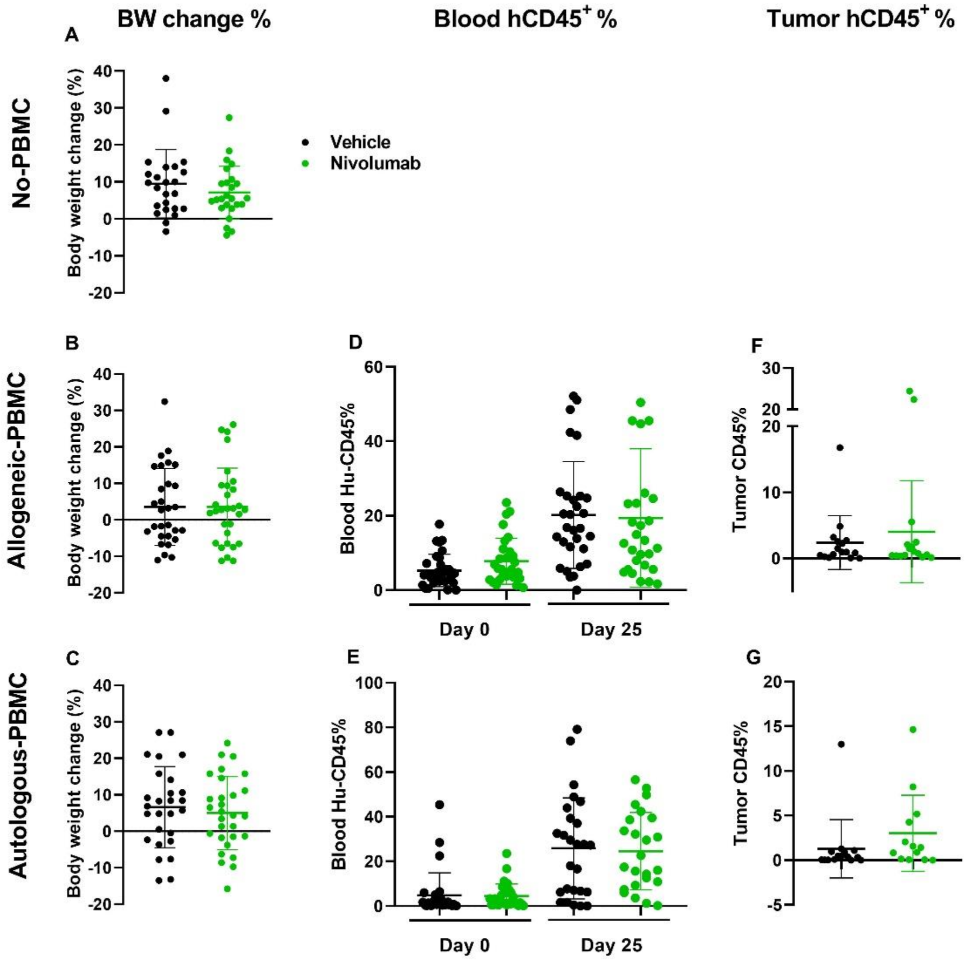

Supplemental Figure 2. **Body weight changes and human immune cell percentage in blood and tumors of mice**

(**A, B, and C**) Body-weight changes in mice under non-humanized (**A**), allogeneic (**B**), and autologous (**C**) conditions. (**D and E**) hCD45$^+$ cell percentages in peripheral blood before and after treatment under allogeneic (**D**) and autologous (**E**) conditions. (**F and G**) Tumor immune infiltrates after anti-PD-1 therapy under allogeneic (**F**) and autologous (**G**) conditions.

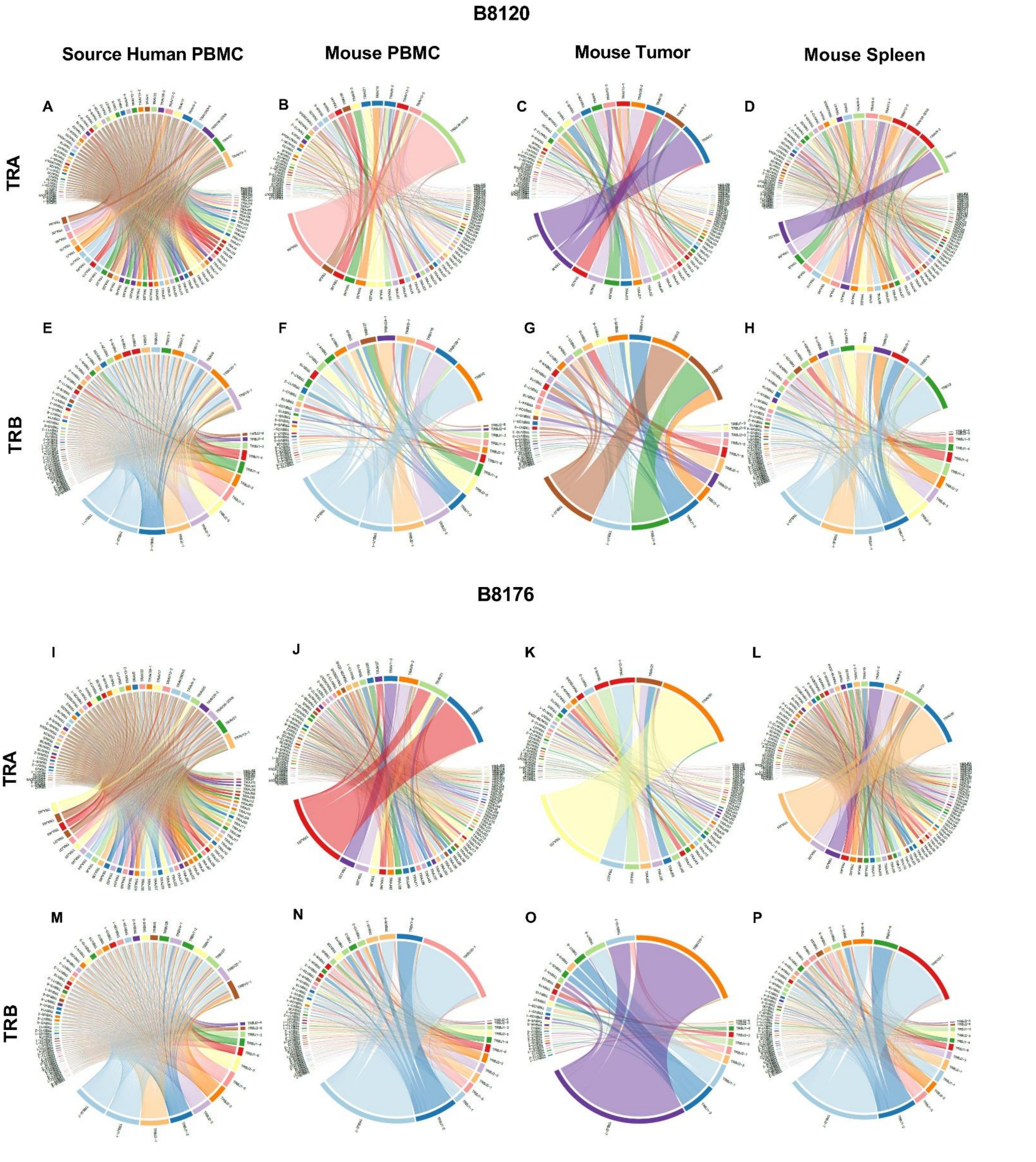

Supplemental Figure 3. **Circus plots of clonal rearrangement patterns**

(**A-D**) The rearrangement patterns for the TCR chains within the TRA clones in model B8120. (**E-H**) The rearrangement patterns for the TCR chains within the TRB clones in model B8120. (**I-L**) The rearrangement patterns for the TCR chains within the TRA clones in model B8176. (**M-P**) The rearrangement patterns for the TCR chains within the TRB clones in model B8176.

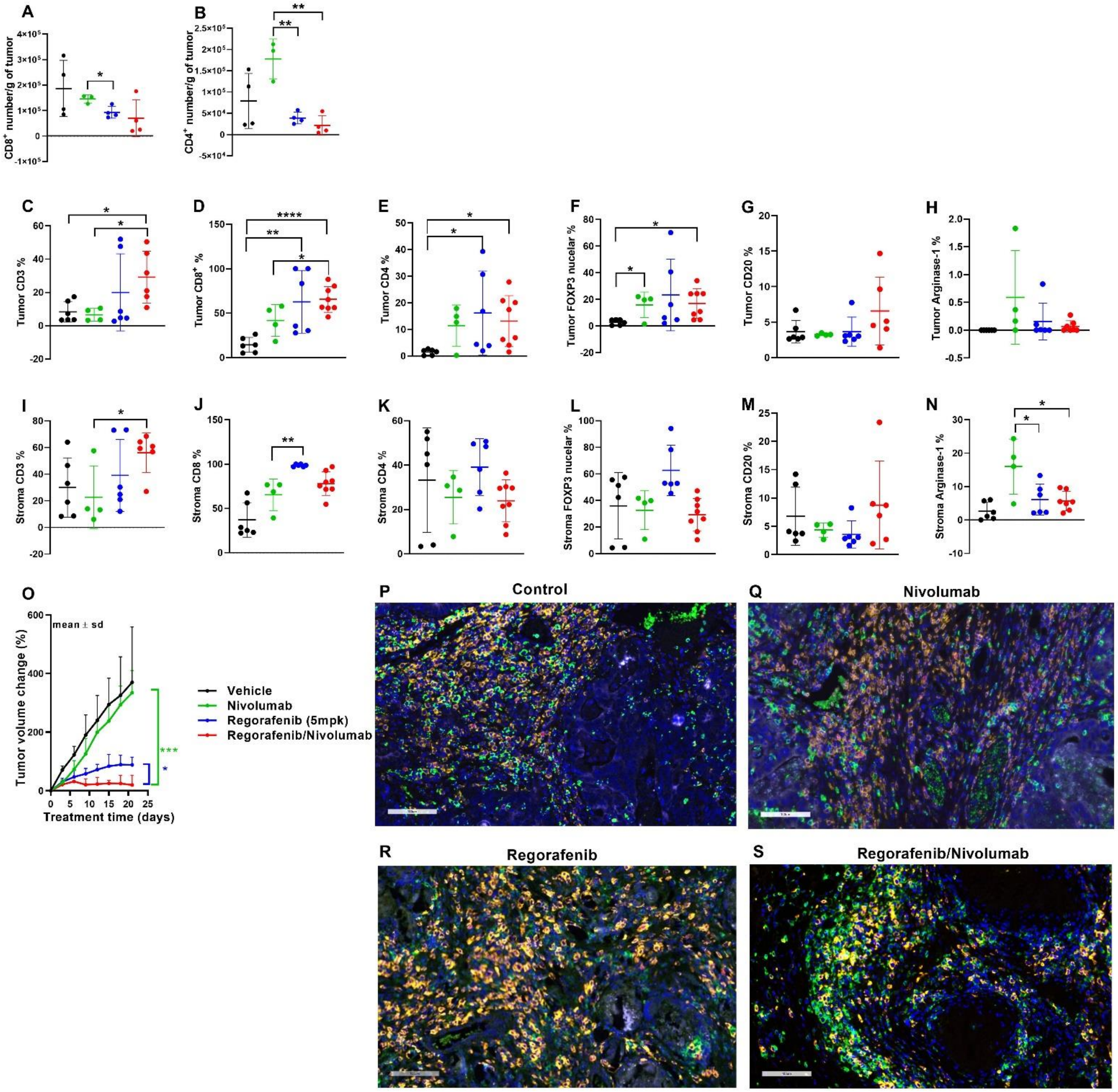

Supplemental Figure 4. Results of anti-PD-1 and regorafenib combination therapy for MSS CRC model

(**A and B**) CD8$^+$ (**A**) and CD4$^+$ (**B**) T-cell numbers per gram of tumor weight. (**C-F**) Cell percentage distributions within the intratumoral compartments. (**I-L**) Cell percentage distributions within the stromal compartments. (**G and M**) CD20$^+$ B-cell distributions within the intratumoral (**G**) and stromal (**M**) compartments. (**H and N**) Arginase-1$^+$ macrophage distribution within the intratumoral (**H**) and stromal (**N**) compartments. (**O**) The effect of the combination of anti-PD-1 therapy and a reduced regorafenib dose on tumor volume change in the C1211 MSS CRC PDX model. (**P-S**) Multiplex immunofluorescence representative images of control (**P**), nivolumab (**Q**), regorafenib (**R**), and regorafinib+nivolumab (**S**) treated tumors showing staining for CD8 (green), CD4 (gold), FOXP3 (red), TUMOR (periwinkle/white). All *P* values ≤ 0.05 were represented as *, *P* values ≤ 0.01 were represented as **, *P* values ≤ 0.001 were represented as ***, and *P* values ≤ 0.0001 were represented as ****.

Supplemental Table 1A: Flow cytometry antibodies

Antibody target	Conjugate	Clone	Company
CD45	PerCP	30-F11	BD Biosciences
CD45	APC	HI30	BD Biosciences
CD3	PE	UCHT1	BD Biosciences
CD4	Alexa 700	RPA-T4	BD Biosciences
CD8	PE-Cy™7	RPA-T8	BD Biosciences
FoxP3	Horizon V450	259D/C7	BD Biosciences
CD56	BB700	B159	BD Biosciences
CD56	Horizon V450	B159	BD Biosciences
CD19	FITC	HIB19	BD Biosciences
CD15	PerCP-Cy5.5	HI98	BD Biosciences
CD14	PE-Cy7	MphiP9	BD Biosciences
CD11b/Mac-1	APC-Cy7	ICRF44	BD Biosciences
CD209	PE	DCN46	BD Biosciences
CD163	PE-CF594	GHI/61	BD Biosciences

Supplemental Table 1B: mIF antibodies

Antibody target	Panel	Clone	Company
Cleaved caspase3	Tumor microenvironment panel	40714	Biocare
Ki-67	Tumor microenvironment panel	SP6	Abcam
Vimentin	Tumor microenvironment panel	D21H3	Cell Signaling Technologies
Pan Cytokeratin	Tumor microenvironment panel	1712R	Bioss
CD8	T cell panel	NBP2-29475	Novus
CD4	T cell panel	BSB5151	BioSB
FoxP3	T cell panel	98377	Cell Signaling Technologies
Arginase-1	Macrophage panel	PA5-85267	ThermoFisher
CD163	Macrophage panel	EPR19518	Abcam
IBA-1	Macrophage panel	EPR16589	Abcam
Ku80	Macrophage panel	2180	Cell Signaling Technologies

Supplemental Table 2. TCR sequencing data (excel sheet attached separately)

Supplemental Table 3. CRC PDX model details (excel sheet attached separately)
